# A Phase Ib Study of the Combination of Personalized Autologous Dendritic Cell Vaccine, Aspirin, and Standard of Care Adjuvant Chemotherapy Followed by Nivolumab for Resected Pancreatic Adenocarcinoma—A Proof of Antigen Discovery Feasibility in Three Patients

**DOI:** 10.3389/fimmu.2019.01832

**Published:** 2019-08-08

**Authors:** Michal Bassani-Sternberg, Antonia Digklia, Florian Huber, Dorothea Wagner, Christine Sempoux, Brian J. Stevenson, Anne-Christine Thierry, Justine Michaux, HuiSong Pak, Julien Racle, Caroline Boudousquie, Klara Balint, George Coukos, David Gfeller, Silvia Martin Lluesma, Alexandre Harari, Nicolas Demartines, Lana E. Kandalaft

**Affiliations:** ^1^Ludwig Institute for Cancer Research, University of Lausanne, Lausanne, Switzerland; ^2^Department of Oncology, Centre Hospitalier Universitaire Vaudois, University of Lausanne, Lausanne, Switzerland; ^3^Institute of Pathology, Centre Hospitalier Universitaire Vaudois, University of Lausanne, Lausanne, Switzerland; ^4^SIB Swiss Institute of Bioinformatics, Lausanne, Switzerland; ^5^Department of Visceral Surgery, Centre Hospitalier Universitaire Vaudois, University of Lausanne, Lausanne, Switzerland

**Keywords:** pancreatic adenocarcinoma, dendritic cell vaccine, antigen discovery, neoantigen, cancer immunotherapy

## Abstract

Despite the promising therapeutic effects of immune checkpoint blockade (ICB), most patients with solid tumors treated with anti-PD-1/PD-L1 monotherapy do not achieve objective responses, with most tumor regressions being partial rather than complete. It is hypothesized that the absence of pre-existing antitumor immunity and/or the presence of additional tumor immune suppressive factors at the tumor microenvironment are responsible for such therapeutic failures. It is therefore clear that in order to fully exploit the potential of PD-1 blockade therapy, antitumor immune response should be amplified, while tumor immune suppression should be further attenuated. Cancer vaccines may prime patients for treatments with ICB by inducing effective anti-tumor immunity, especially in patients lacking tumor-infiltrating T-cells. These “non-inflamed” non-permissive tumors that are resistant to ICB could be rendered sensitive and transformed into “inflamed” tumor by vaccination. In this article we describe a clinical study where we use pancreatic cancer as a model, and we hypothesize that effective vaccination in pancreatic cancer patients, along with interventions that can reprogram important immunosuppressive factors in the tumor microenvironment, can enhance tumor immune recognition, thus enhancing response to PD-1/PD-L1 blockade. We incorporate into the schedule of standard of care (SOC) chemotherapy adjuvant setting a vaccine platform comprised of autologous dendritic cells loaded with personalized neoantigen peptides (PEP-DC) identified through our own proteo-genomics antigen discovery pipeline. Furthermore, we add nivolumab, an antibody against PD-1, to boost and maintain the vaccine's effect. We also demonstrate the feasibility of identifying personalized neoantigens in three pancreatic ductal adenocarcinoma (PDAC) patients, and we describe their optimal incorporation into long peptides for manufacturing into vaccine products. We finally discuss the advantages as well as the scientific and logistic challenges of such an exploratory vaccine clinical trial, and we highlight its novelty.

## Introduction

Pancreatic ductal adenocarcinoma (PDAC) is the seventh leading cause of cancer-related death in the world in 2018 (1), with an overall 5-year survival rate of ~5% (2). Approximately 70% of deaths are due to widespread metastasis and the remaining cases have limited metastasis but extensive primary tumors which eventually lead to mortality (3). Surgery is the only potential hope of cure for PDAC, but tumors are resectable only in 20% of patients at the time of diagnosis. Therapeutic research efforts have mainly focused on improvements in radio/chemo treatments and to date, there are only a few chemotherapeutic agents that have shown to be effective against advanced pancreatic cancer, including gemcitabine with or without abraxane (4). At present, it is difficult to conclude that there is a definite SOC adjuvant chemotherapy for all patients with PDAC. However, multiagent adjuvant therapy (modified folforinox) has been demonstrated to be more effective than gemcitabine alone in the adjuvant setting, but its use is limited only to patients with excellent performance status (5). Recently it has suggested that gemcitabine plus capecitabine is a valid option for these patients since it has been shown that it is more efficient than gemcitabine alone (6).

One of the most promising new cancer treatment approaches is immunotherapy. Recent studies have shown that PDAC is an immunogenic tumor. Antigens expressed on pancreatic tumor cells able to induce specific B and T cells comprise (7): Wilms' tumor gene 1 (WT1) (75%) (8), mucin 1 (MUC1) (over 85%) (9), human telomerase reverse transcriptase (hTERT) (88%) (10), mutated K-RAS (nearly 100%), survivin (77%), carcinoembryonic antigen (CEA) (over 90%) (11), HER-2/neu (over 60%) (12), p53 (over 65%) (13), and α-enolase (ENO1) (14). Several studies have reported that dysfunction of the immune system is one of the key contributors for the development of PDAC (15, 16). Moreover, PDAC is known to have an immunosuppressive tumor microenvironment characterized by (i) the absence of intratumoral effector T-cells (17, 18), (ii) the presence of an inflammatory tumor micro-environment led by the RAS oncogene (19), and (iii) massive infiltration of immunosuppressive leukocytes into the tumor microenvironment, which predicts poor survival (18, 20, 21). Additionally, the analysis of immune infiltrates in human tumors has demonstrated a positive correlation between prognosis and the presence of humoral response to pancreatic antigens (MUC-1 and mesothelin) (22, 23) or of tumor-infiltrating T cells (20, 24). Therefore, cancer immunotherapy can be a promising alternative treatment for PDAC patients.

A major mechanism of immune resistance engaged by tumors is the enforcement of immune checkpoint pathways, aiming to shutdown T cells specific for tumor antigens. An important immune checkpoint is mediated by the programmed cell death protein 1 (PD-1) expressed on the surface of activated T cells during initial activation (25, 26). The major role of PD-1 is to limit the activity of T cells in peripheral tissues at the time of an inflammatory response to infection and to restrict autoimmunity (27, 28). Cancer immunotherapy targeting anti-PD-1 (e.g., nivolumab, pembrolizumab), as well as anti-cytotoxic T-lymphocyte-associated antigen 4 (anti-CTLA-4, ipilimumab), has changed the treatment landscape of several tumors (29). Yet the success of immunotherapy has not been proven effective for the treatment of metastatic pancreatic cancer patients (30), who have been shown unresponsive except for the population with mismatch-repair deficiency which comprises only 0.8% (31). A broad array of clinical trials in pancreatic cancer have been completed or are ongoing using different combinations with ICB (32, 33). However, the most adequate combination for PDAC patients is not clear so far.

Dendritic cell (DC)-based vaccines for cancer immunotherapy have been studied and tested for more than a decade and proven clinically safe and efficient to induce tumor-specific immune responses, however only limited efficacy was observed in patients with advanced recurrent disease after DC vaccination (34, 35). Several groups have attempted to test safety and efficacy of DC-based vaccines against pancreatic cancer in early phase clinical trials, loading DCs with tumor associated antigens (TAAs) *ex vivo*, and subsequently re-infusing them in patients, yet with low clinical benefit so far (36–39). One possible reason for reduced vaccine efficacy could be that most cancer vaccines tested to date were targeted against defined non-mutated self-antigens. Tumors express two major kinds of antigens that can be recognized by T cells: non-mutated self-antigens and mutated neoantigens, generated in tumor cells due to their inherent genetic instability (40). Tumor cells usually harbor between 10 and few thousands private somatic mutations, as identified by deep sequencing analysis, and even among tumors of the same histotype, most mutations are different (41, 42). Thus, neoantigens are mostly “private” and patient-specific (43) and trigger a higher more robust T-cell response. Indeed, increasing evidence associates clinical benefit from immunotherapy with specific responses to private tumor epitopes (44–48), leading to increased interest in neoantigen vaccination (40).

Several clinical trials describing vaccines designed to harness neoantigen-specific immunity have been recently reported mainly in melanoma patients: The first study reported the feasibility, safety and efficacy of a DC vaccine pulsed with neoantigen peptides (49). Another phase I study has evaluated a peptide vaccine targeting up to 20 predicted personal tumor neoantigens and demonstrated an expansion of the repertoire of neoantigen-specific T cells which correlated with clinical benefit (50). A second group performed a phase I study using RNA vaccines that contained up to 10 mutations per patient and demonstrated that these vaccines can mobilize specific anti-tumor immunity against these cancers (51). These studies provide proof-of-principle that a personalized vaccine can be produced and administered to a patient to generate highly specific immune responses against that individual's tumor, showing that a personalized neoantigen vaccine broadens the repertoire of neoantigen-specific T cells substantially beyond what is induced by existing immunotherapeutics.

To determine whether targetable mutations and neoantigens exist in PDAC, several studies have been performed using genomic profiles of PDAC tumor samples. A whole-genome sequencing and copy number variation (CNV) analysis was performed on 100 pancreatic ductal adenocarcinomas (PDACs) and found in total 11,868 somatic structural variants at an average of 119 per individual (range 15–558) (52). Furthermore, the genomic profile of 221 PDAC tumors were analyzed and the findings revealed that nearly all PDAC samples harbor potentially targetable neoantigens (53). To define the importance of neoantigens in PDAC, one study compared stage-matched cohorts of treatment-naive, surgically resected, rare long-term survivors to short-term survivors with a more typical poor outcome. The authors detected a median of 38 predicted neoantigens per tumor, and showed that the association of higher neoantigen quantity and CD8^+^ T-cell infiltrate with survival was independent of adjuvant chemotherapy, suggesting that neoantigen quality, and not purely quantity, correlates with survival (54).

We hypothesize that effective vaccination in PDAC patients along with interventions that can reprogram important immunosuppressive factors in the tumor microenvironment can enhance tumor immune recognition, thus enhancing response to PD-1/PD-L1 blockade. To this end, we designed a phase 1b trial where we incorporated a vaccination schedule of a novel autologous DC pulsed with personalized neoantigen peptides (PEP-DC) identified through our own proteo-genomics antigen discovery pipeline in the SOC chemotherapy adjuvant setting followed by nivolumab. We hereby set the objectives and design of our study, and we demonstrate the feasibility of identifying personalized neoantigens in three PDAC patients, and their optimal incorporation into long peptides for manufacturing into vaccine products.

## Materials and Methods

### Clinical Study Design

This is a phase Ib trial (CHUV-DO-0017_PC-PEPDC_2017) to evaluate the feasibility, safety, immunogenicity, and efficacy of subcutaneous DC vaccine loaded with personalized peptides (PEP-DC), in combination with SOC chemotherapy (gemcitabine/capecitabine) and enteric-coated aspirin, followed by the anti-PD-1 antibody nivolumab to boost and maintain the vaccine's effect in patients with surgically resected PDAC. The components of the vaccine to be investigated in this study include agents for which safety has been previously demonstrated to be acceptable. This trial has been approved by Swissmedic and the competent Ethics Committee. Before any study-specific procedure is performed, a signed and dated informed consent is obtained. In order to be eligible, patients must present: (a) histologically confirmed resected adenocarcinoma of the pancreas (T1–T4, N 0–1, minimum 2 cm–AJCC 8th ed.) and (b) appropriate amount of tumoral tissue collected from the cytoreductive surgery, allowing the identification of top 10 personalized peptides (PEP) for preparation of PEP-DC vaccine.

#### Objectives

The primary objectives of the trial are to determine: (1) the feasibility of producing and administering PEP-DC vaccine in the indicated patient population; (2) the safety and tolerability of the study treatment vaccine and aspirin given together with SOC chemotherapy, and followed by nivolumab; (3) the immunogenicity by measuring acquired T cell mediated immune activation events post vaccination. This study has also a secondary objective, which is to evaluate relapse free survival at 6, 12, 18, 24, and 36 months and overall survival in the indicated population of patients.

#### Statistical Methods

We hypothesize that the delivery of the PEP-DC vaccine through the subcutaneous route in combination with aspirin, nivolumab, and adjuvant chemotherapy in advanced pancreatic cancer patients is feasible, safe without additional toxicity, and immunogenic. Based on study feasibility and anticipated accrual rate, a total of 12 evaluable patients is expected to enter this Phase Ib study if treatment limiting toxicities (TLTs) are in the acceptable range.

The feasibility hypothesis for PEP-DC vaccine will be assessed by (a) the number of patients in which vaccine production is successful (at least 6 doses are manufactured and released), and (b) the number of patients who receive at least one dose of PEP-DC vaccine (since the mainstay of the therapeutic approach here is PEP-DC) and the corresponding percentages in the ITT population (i.e., all registered patients). Exact binomial confidence intervals for the corresponding rates will be estimated.

The safety and tolerability of the PEP-DC vaccine in combination with other protocol drugs will be evaluated by the occurrence of TLTs and adverse events (AEs) in both the “TLT evaluation” and the safety population. The severity of toxicities will be classified according to the NCI CTCAE Version 4.03 and will be presented in tabular as well as graphical format. For each patient, each AE will be presented considering the highest (worst) grade of toxicity observed over the whole treatment period according to CTCAE version 4.03. Although the safety of the vaccination backbone has been already established, a continuous monitoring rule will be followed, to allow for early termination of the study. Any patient who receives at least one vaccination will be included in the toxicity (safety) analysis. A Bayesian rule will be employed to monitor TLTs after groups of 4 patients have been treated and complete the final TLT evaluation.

As only 5 weeks are allocated for target prioritization, assessment of pre-vaccination immune responses against the predicted neoantigens will not be performed to assist their selection. Therefore, the selection of long peptides is done *in silico* by the NeoDisc pipeline. The immunogenicity of PEP-DC vaccine will be assessed (based on ITT population as well as the safety population) by measuring acquired, T cell-mediated immune activating events post vaccination compared to pre-vaccination levels. Descriptive statistics of absolute and relative differences will be calculated overall and for subgroups of interest.

#### Regimen

The study was designed so that eligible subjects with PDAC who undertook cytoreductive surgery followed by chemotherapy may plan to enroll in the vaccine study. Should the subject wishes, and upon informed consent, tissue can be harvested at the time of surgery for identification of personalized targets for vaccination. Screening of patients may be completed after the collection of tumor was performed during the surgery. Upon registration for the trial, all patients would receive 8 cycles of 21 day cycle of gemcitabine/capecitabine. Eligible patients will undergo apheresis during the last week of the third cycle of gemcitabine/capecitabine to collect peripheral blood mononuclear cells for DC vaccine production. Patients will receive at least six PEP-DC vaccinations starting concomitant with the 5th cycle of chemotherapy. PEP-DC vaccine of 5–10 × 10^6^ autologous DC in 1 ml volume/treatment will be delivered subcutaneously every 3 weeks. Patients will receive oral enteric-coated aspirin daily for the duration of the study starting from the day of first vaccination until the end of study. Nivolumab will be administered starting 3 weeks after last chemotherapy cycle and will be given during the vaccination period until the last vaccine dose. Afterwards, it will be given as a maintenance therapy until appearance of new lesion(s) or unacceptable toxicity for maximum 2 years.

To verify that the combination of PEP-DC vaccine and enteric-coated aspirin during and following standard adjuvant chemotherapy, followed by nivolumab, will significantly enhance tumor immunogenicity, and allow tumor response, the translational objectives of the study are the following: (a) to deeply characterize the tumor microenvironment of pancreatic adenocarcinoma patients; (b) to assess the overall effects of the combined PEP-DC vaccine during and following standard adjuvant chemotherapy, followed by nivolumab on peripheral blood and plasma; (c) to determine tumor antigens against which the treatment elicits a response.

### Identification of Personalized Targets for Vaccination With NeoDisc

#### Processing of Patients' Material for PEP-DC Vaccine Preparation

Informed consent of the participants was obtained following requirements of the institutional review board (Ethics Commission, CHUV). The translational research has been approved by the CHUV ethics committee (protocols 2017-00305).

#### DNA Extraction and Sequencing

DNA was extracted for HLA typing and exome sequencing with the commercially available DNeasy Blood & Tissue Kit (Qiagen, Hilden, Germany), following manufacturers' protocols. Five hundred nanograms of gDNA were used to amplify HLA genes by PCR. High resolution 4-digit HLA typing was performed with the TruSight HLA v2 Sequencing Panel from Illumina on a MiniSeq instrument (Illumina) (Supplementary Table 1). Sequencing data were analyzed with the Assign TruSight HLA v2.1 software (Illumina). For exome sequencing, SureSelect Exome V5 library type (Sureselect v5 capture, Agilent Technologies, Santa Clara, CA, USA), and paired end reads were chosen, with at least 100x coverage for the tumor and PBMCs.

#### LC-MS/MS Analyses of Eluted HLA Peptides

For immunoaffinity purification of HLA peptides from tissues, we applied a previously published protocol (55, 56). Briefly, anti-HLA-I and anti-HLA-II monoclonal antibodies were purified from the supernatant of HB95 (ATCC® HB-95™) and HB145 cells (ATCC® HB-145™) using protein-A sepharose 4B beads (Invitrogen, Carlsbad, California), and cross-linked to the beads. Snap-frozen PDAC tissue samples were homogenized in lysis buffer on ice in 3–5 short intervals of 5 s each using an Ultra Turrax homogenizer (IKA, T10 standard, Staufen, Germany) at maximum speed, as previously described (55, 56). Lysates were cleared by centrifugation at 25,000 rpm (Beckman Coulter, JSS15314, Nyon, Switzerland) at 4°C for 50 min. The Waters Positive Pressure-96 Processor (Waters, Milford, Massachusetts) was used with 96-well, 3 μm glass fiber and 10 μm polypropylene membranes micro-plates (Seahorse Bioscience, North Billerica, Massachusetts). A depletion step of endogenous antibodies was performed with plates containing Protein-A beads, and then the lysates were passed through a plate containing beads cross-linked to anti-HLA-I, and then sequentially through a plate with the anti-HLA-II cross-linked beads. After washing with varying concentrations of salts, the beads were washed twice 2 mL of 20 mM Tris-HCl pH 8. HLA complexes and the bound peptides were eluted directly into pre-conditioned Sep-Pak tC18 100 mg plates (ref number: 186002321, Waters) with 1% TFA. After washing the C18 wells with 2 mL of 0.1% TFA, HLA-I peptides were eluted with 28% ACN in 0.1% TFA, and HLA-II peptides were eluted from the class II C18 plate with 500 μL of 32% ACN in 0.1% TFA. HLA-I, and HLA-II peptide samples were dried using vacuum centrifugation (Concentrator plus Eppendorf) and stored at −20°C.

We measured the peptides with LC-MS/MS system consisting of an Easy-nLC 1200 (Thermo Fisher Scientific, Bremen, Germany) and the Q Exactive HF-X mass spectrometer (Thermo Fisher Scientific, Bremen, Germany). Peptides were separated on a 450 mm analytical column of 75 μm inner diameter for 120 min using a gradient of H2O/FA 99.9/0.1% (A) and ACN/FA 80/0.1% (B). The gradient was run as follows: 0 min 2% B, then to 5% B at 5 min, 35% B at 85 min, 60% B at 100 min, and 95% B at 105 min at a flow rate of 250 nL/min.

MS spectra were acquired in the Orbitrap from m/z = 300–1,650 with a resolution of 60,000 (m/z = 200), ion accumulation time of 80 ms. The AGC was set to 3e6 ions. MS/MS spectra were acquired in a data-dependent manner, and 10 most abundant precursor ions were selected for fragmentation, with a resolution of 15,000 (m/z = 200), ion accumulation time of 120 ms and an isolation window of 1.2 m/z. The AGC was set to 2e5 ions, dynamic exclusion to 20 s, and a normalized collision energy (NCE) of 27 was used for fragmentation.

#### NeoDisc Pipeline

##### Alignment

Exome sequence reads were aligned to the Genome Reference Consortium Human Build 37 assembly (GRCh37) with BWA-MEM version 0.7.17 (57). The resulting SAM format was sorted by chromosomal coordinate and converted into a BAM file, then PCR duplicates were flagged, using the Picard AddOrReplaceReadGroups and MarkDuplicates utilities, respectively (from http://broadinstitute.github.io/picard). Various quality metrics were assessed with the Picard MarkDuplicates, CollectAlignmentSummaryMetrics, and CalculateHsMetrics utilities. Following GATK best practices, GATK BaseRecalibrator (within GATK v3.7-0) was used to recalibrate base quality scores (BSQR) prior to variant calling (58, 59). BQSR corrects base quality scores based on an estimation of empirical error frequencies in the alignments. The recalibrated tumor and germline BAM files were then used as input for each of three variant callers: GATK HaplotypeCaller; MuTect v1; and VarScan 2.

##### Caller 1: GATK HaplotypeCaller

The GATK HaplotypeCaller algorithm improves variant calling by incorporating *de-novo* assembly of haplotypes in variable regions, thus reducing the overall false-positive variant call rate (58, 59). HaplotypeCaller was run in GVCF mode on each tumor and germline recalibrated BAM file to detect SNV and Indel variants. The resultant gVCF files were combined using GATK GenotypeGVCF to produce raw variant calls for tumor and germline within in a single VCF. Subsequent variant quality score recalibration, following GATK best practices, was performed separately for SNVs and Indels (insertions/deletions) using the GATK variant Recalibrator tool to identify high-confidence calls. Variant quality was assessed by the GATK VariantEval tool. Patient-specific SNPs were defined as variants present in both tumor and germline, while variants present only in tumor were defined as somatic mutations.

##### Caller2: MuTect v1

The MuTect variant calling algorithm predicts somatic mutations based on log odds scores of two Bayesian classifiers (from https://github.com/broadinstitute/mutect). The first classifier identifies non-reference variants in the tumor sample while the second detects whether those variants are tumor specific. Candidate somatic mutations are then filtered based on read support, for example by ensuring that supporting reads map to both DNA strands, in order to reduce next-generation sequencing artifacts. Identified somatic mutations are exported in VCF format.

##### Caller3: VarScan 2

The VarScan2 algorithm, unlike GATK and MuTect, relies on hard filtering of calls rather than Bayesian statistics (60). This has the advantage of being less sensitive to bias such as extreme read coverage and sample contamination. VarScan 2 filters reads based on parameters such as read quality, strand bias, minimum coverage, and variant frequency. The multisample pileup file required for VarScan 2 input was generated with SAMtools (61, 62). VarScan 2 was run using default parameters and generated a VCF containing SNVs and Indels for both somatic mutations and SNPs.

##### Non-redundant call set

Variant calls from GATK, MuTect v1, and VarScan 2 were combined into a single VCF that contains the union of the variants of all three callers. Ambiguous calls (i.e., different calls at the same genomic coordinate) were resolved by a simple majority rule. If there was no majority, the call was rejected. GATK ReadBackedPhasing was used to retrieve the phasing information of all variants in the combined VCF (58, 59). The functional effect of the variants was annotated by SnpEff which predicts the effects of variants on genes based on reference databases. To maximize variant annotation we used annotations from the hg19 (Refseq) and GRCH37.75 (Ensembl) databases (63–65). This non-redundant, annotated VCF file was used for further genomics and proteogenomics analyses.

#### Prediction and Prioritization of Neoantigens

For the identification of neoantigens, only “high confidence” calls were selected, defined as the set of variants containing all somatic mutations plus linked SNPs (i.e., those SNPs present on the same allele as the somatic mutation) detected by MuTect v1 alone or by a combination of at least two of the three variant callers described above. The novel amino acid generated by each single nucleotide somatic mutation was placed at the center of a 31mer peptide that also included any amino acid changes resulting from non-synonymous linked SNPs. In the case of a somatic indel mutation, the entire polypeptide encoded by the new open-reading frame plus the upstream 24 amino acids could be subjected to HLA ligand prediction. However, for the described three PDAC samples this option was disabled.

HLA-I and HLA-II ligands were predicted by the MixMHCpred.v2.0.2 and MixMHC2pred.v1 algorithms, respectively (66–68). Both algorithms have been trained on naturally presented peptides and compute the likelihood of a peptide to bind to one of a given set of HLA alleles. Mutant peptides of sizes ranging from 9 to 12 and 12 to 19 amino acids, derived from the 31mer were supplied as input for HLA-I and HLA-II predictions, respectively, using patient-specific allotypes as determined by HLA typing (Supplementary Table 1).

Tissue-specific gene expression data was downloaded from The Genotype-Tissue Expression (GTEx) project, a public resource that contains data from 53 non-diseased tissues across nearly 1,000 individuals (69). We used a custom R script to retrieve gene expression values, based on GTEx v7 publicly available data. The 90th percentile expression of the wild type gene in the tissue-derived tumor was reported from GTEx data, and mutations in genes not expressed (TPM < 1) in pancreas were excluded.

Due to the intrinsic content and properties of protein sequences, HLA ligands are not distributed equally along proteins and tend to cluster in hotspots. We captured this information across dozens of cell types in our ipMSDB database (70). The overlap of the wild-type-form of a mutant peptide with a hotspot in ipMSDB was calculated, as well as the level of presentation of the source protein. Any mutant peptide matching any wild-type sequence in SwissProt (71) or found in the reference GRCh37 (64) proteome was filtered out.

Finally, we used a custom python script to design the best long peptide(s) for every mutation, encompassing the highest possible number of HLA-I and HLA-II binding peptides (MixMHCpred and MixMHC2pred %Rank < 5% or found in ipMSDB). Long peptides were ranked by the minimum *p*-value of the predicted HLA-I neoantigens, and the top 10 long peptides were selected.

#### Proteogenomics

For every sample, we created a reference fasta file where residue mutation information was added to the header of the affected translated transcripts, in a format compatible with MaxQuant v1.5.9.4i as previously reported (72). We used the GENCODE v24 (73) (GRCh37 human reference assembly, downloaded from https://www.gencodegenes.org/human/release_24lift37.html) as the standard reference dataset (89,543 entries). We parsed the GENCODE comprehensive gene annotation file, in GFF3 format, to extract genomic coordinate information for every exon. These coordinates were compared with sample-specific variant coordinates to derive non-synonymous amino acid changes within each protein.

For every patient, we searched the immunopeptidomics MS data against the patient-specific customized reference database, including a list of 247 frequently observed contaminants. The enzyme specificity was set as unspecific, and peptides with a length between 8 and 25 AA were allowed. The second peptide identification option in Andromeda was enabled. A false discovery rate (FDR) of 5% was required for peptides and no protein FDR was set. The initial allowed mass deviation of the precursor ion was set to 6 ppm and the maximum fragment mass deviation was set to 20 ppm. Methionine oxidation and N-terminal acetylation were set as variable modifications.

### PEP-DC Manufacturing

The PEP-DC vaccine is composed of autologous monocyte-derived DC pulsed with personalized peptides (PEP). Monocytes are enriched from a fresh leukapheresis using CD14^+^ cells selection on the CliniMACS Prodigy (Miltenyi). This process is GMP compliant and allows for a fast and reliable monocyte selection in a closed system. Purified monocytes are differentiated into immature monocyte-derived DC (iDC) by a 5 days culture in the presence of IL-4 and GM-CSF. On day 6, iDC are then loaded overnight with 10 long peptides and matured/activated for 6–8 h using a maturation cocktail composed of MPLA and IFNγ. Cells are finally harvested and cryopreserved as vaccine doses (5–10 × 10^6^ cells per dose). For each injection of PEP-DC vaccine, one dose is thawed, washed and resuspended in NaCl 0.9% supplemented with 1% human albumin before being transferred into syringes and stored at 2–8°C until administration.

### Immunogenicity Assessment of PEP-DC Candidates Pre-immunization

The immunogenicity the long peptides was evaluated in cryopreserved peripheral blood mononuclear cells (PBMC) from the three subjects as described (74). PBMC were thawed, rested overnight in RPMI 10% FBS with Penicillin/Streptomycin. For the *in vitro* stimulation (IVS), cells were plated in 24- to 96-well plates at 2 × 10^6^ cells per well in RPMI, 8% human serum supplemented with Penicillin/Streptomycin, 50 μM beta-mercaptoethanol and recombinant human IL-2 at a final concentration of 100 UI/ml. The cells were stimulated with peptide pools containing 1 μg/ml of each candidate peptide. At day 12, intracellular cytokine stainings (ICS) were performed. Each individual well was splitted in two identical fractions and one fraction only was re-challenged with 1 μg/ml of the corresponding peptide for 16–18 h at 37°C and 5% CO2 in presence of 1 μg of brefeldin A (Golgiplug, BD). As a positive control, cells stimulated with staphylococcal enterotoxin B (SEB) at a concentration of 0.25 ng/ml. After 16–18 h of re-stimulation with individual long peptides, cells were harvested and stained with anti-CD3, anti-CD8, anti-CD4, anti-IL-2, anti-TNF-α, anti-IFN-γ (BD biosciences), and with viability dye (Life technologies). Flow cytometry was performed using a four-lasers Fortessa (BD biosciences) and analyzed with FlowJo v10 (TreeStar).

## Results

**Figure 1:**
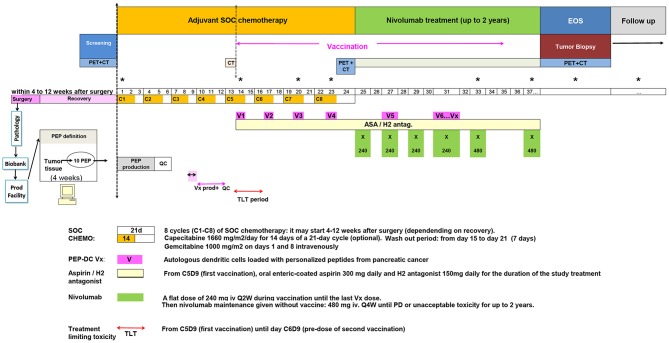
Clinical trial study design.

We here present a novel study were a vaccination schedule is incorporated in the SOC chemotherapy adjuvant setting in patients with non-metastatic resectable pancreatic adenocarcinoma followed by nivolumab (an antibody against PD-1), to boost and maintain the vaccine's effect (Figure 1). The study was optimally designed to offer innovative cancer vaccines for a PDAC patient population that on one hand would fit the course of standard of care, and on the other hand will be feasible in terms of the time required for the process of antigen discovery and the manufacturing of the vaccine.

It has been correctly pointed out that putting a mutanome-based individualized treatment concept into practice requires both highly interdisciplinary research and an innovative drug development process (75). To fit the tight schedule of the clinical trial, a period of 5 weeks was dedicated for antigen discovery. Upon reception of a pair of tumor tissue sample and matched PBMCs, DNA extraction is performed for whole exome sequencing and HLA typing, and the tumor tissue sample is processed for purification of HLA-I and HLA-II peptides for MS analyses. Within the 2 weeks required for sequencing the DNA samples, HLA typing and MS analyses are completed. The following 2 weeks are dedicated for executing the NeoDisc pipeline, for data mining and for manual inspection of the data and results, leading to the selection of 10 long optimally designed neoantigens. Finally, the production needs to be “on demand,” cost-effective, rapid, and compliant with Good Manufacturing Practice (GMP).

### NeoDisc Pipeline for Neoantigen Discovery in PDAC

**Figure 2:**
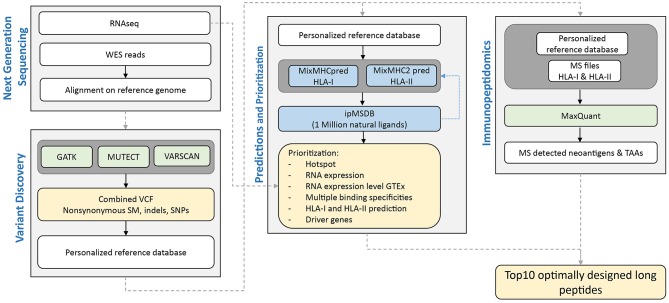
Schematic overview of NeoDisc pipeline for prioritization of neoantigens in PDAC for the design of optimally long peptides for vaccination.

We here tested the feasibility of prioritizing neoantigens in PDAC as targets for our PEP-DC vaccine in three PDAC patients, 14JQ, 154H, and 16AY. The NeoDisc pipeline integrates multiple types of data input from next generation sequencing data, MS immunopeptidomics datasets, and publicly available resources (Figure 2). First, the NeoDisc pipeline requires a list of non-synonymous somatic mutations that affect protein-coding regions as identified by three different mutation-calling algorithms: MuTect, VarScan2, and GATK. A combined VCF file is generated and annotated with amino acid changes and transcript information. To increase accuracy, only “high confidence” calls were selected, defined as the set of somatic mutations detected by MuTect alone or by a combination of at least two of the three variant callers described above. As expected, the mutational load in the three PDAC patients was low, with 60, 39, and 23, non-synonymous somatic mutations in 14JQ, 154H, and 16AY, respectively, which is within the range previously reported (53). Among them, we detected mutations in predicted driver genes, the MLLT4 (Ser1708Ala) and PTPN12 (Gly532Glu) (76–78). We then attempted to identify personalized neoantigens using two different approaches; direct identification with mass spectrometry and by prediction of HLA ligands encompassing any of these mutations.

We first performed MS immunopeptidomics analyses on exactly the same tumor tissue used for the genomics analysis from the three PDAC patients, and applied a proteogenomics pipeline as previously described (72) in order to identify neoantigens naturally presented in the PDAC tissues. We have identified 11,437, 4,437, and 6,158 HLA-I and 1,569, 448, and 3,319 HLA-II peptides, from the 14JQ, 154H, and 16AY tumor tissues, respectively (Supplementary Table 2). However, no neoantigens could be identified by MS. Either many of the potential neoantigens remain undetected in the MS-based analyses because of the lack of sensitivity, or they might not be naturally presented. The likelihood of detecting neoantigens by discovery MS increases with the overall depth of ligandomic data available and with the mutational load. Here, both aspects were not sufficient to successfully detect neoantigens.

Additional tumor-associated antigen (TAAs) derived HLA ligands are frequently identified by MS, such as “normal” (wild-type) proteins overexpressed or restricted to tumors (e.g., MelanA, Tyrosinase, PMEL in melanoma; NY-ESO in multiple cancer types). Such targets have been exploited in innovative personalized vaccines and T cell based therapies (79, 80). We have identified multiple TAAs in the immunopeptidome for each of the three PDAC patients, including the testis-specific protein bromodomain testis-specific protein (BRDT), L-lactate dehydrogenase C chain (LDHC), outer dense fiber protein 2 (ODF2), coiled-coil domain-containing protein 110 (CCDC110), tumor associated antigens mesothelin (MSLN), mucin-1 (MUC1), prolyl endopeptidase FAP (FAP), and the cellular tumor antigen p53 (TP53) (Supplementary Table 2). Nevertheless, after thorough data mining, we estimated that these ligands were unlikely to be immunogenic and therefore have decided not to include them in the vaccine.

### Prioritization of Neoantigens and Design of Long Peptides

**Figure 3:**
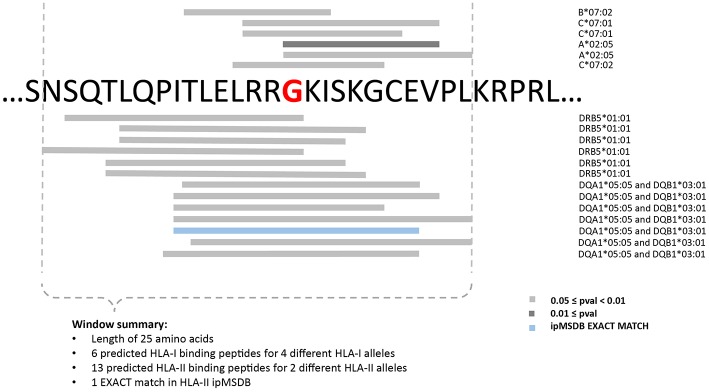
Example for the design of the minimally long peptide covering the mutation Arg288Gly in PFH20 gene identified in 154H PDAC patient.

Consequently, in the three PDAC patients, the selection of targets was based exclusively on prediction of neoantigens. To increase accuracy, the “high confidence” calls were selected, and a list of 31 mer peptides with mutation in the middle position was then generated and subjected to binding predictions of HLA class I (9–12 mers) and class II (12–19 mers) with the MixMHCpred.v2.0.2 and MixMHC2pred.v1 algorithms, respectively (66, 68, 81). Both algorithms have been trained on naturally presented peptides and compute the likelihood of a peptide to bind to one of the given set of the patient HLA alleles. We have previously showed that large scale immunopeptidomics dataset may help in prioritizing predicted neoantigens (70, 82). Therefore, we mapped the list of predicted neoantigens on our ipMSDB ligandomic database that contains a million of HLA-I and HLA-II ligands. The overlap between the predicted neoantigen and the wild-type (WT) form present in ipMSDB was determined, as well as the level of presentation of the source genes. These values were considered for prioritization; neoantigens matching exactly WT counterparts in ipMSDB were prioritized (Table 1). Mutated source genes that were underrepresented in ipMSDB were excluded. In addition, we excluded predicted neoantigens that are identical to other WT sequences in the human proteome (GRCh37 Genome assembly and UniProt database) and all predicted neoantigens derived from highly mutated genes, which are likely to be false positives. Finally, we excluded genes that are known not to be expressed in pancreas (TPM < 1 in GTEx). For each mutation, we designed a few long peptides covering as many predicted HLA-I and HLA-II neoantigens as possible. We ranked the mutations in the format of long peptides according to the best predicted binding affinity to HLA-I alleles (%Rank ≤ 5% MixMHCpred.v2.0.2), the number of HLA-I and HLA-II predicted neoantigens harboring the mutation, and the number of represented HLA alleles. Finally, for each mutation we selected the shortest long peptide covering as many predicted HLA-I and HLA-II neoantigens (Figure 3) and completed the list of ten long peptides (PEP).

**Table 1:** Basic clinical information and detailed information about the 10 optimally designed long peptides for each patient.

**Rank**	**Chromosome position**	**Gene**	**Expression in pancreas, GTEx [TPM]**	**Mutation**	**Gene driver and mutation status**	**Long peptide sequence**	**ipMSDB HLA-I**	**ipMSDB HLA-II**	**Lowest HLA-I binding pval**	**Lowest HLA-II binding pval**	**# predicted peptides**	**# HLA-I alleles**	**# HLA-II alleles**
**14JQ, PDAC, 60 NON-SYNONYMOUS SOMATIC MUTATIONS**													
1	16_8994451	USP7	11.509	p.Tyr749Asp		LYEEVKPNLTERIQDDDVSLDKALDE	EXACT		0.002	0.01209	31	4	3
2	7_27169740	HOXA4	1.06	p.Ala205Thr		VVYPWMKKIHVSTVNPSYNGGEPKRSRT	EXACT	EXACT	0.004	0.0005	58	3	7
3	9_33797978	PRSS3	13983	p.Val175Ile		TLDNDILLIKLSSPAIINSRVSAISLPT	EXACT	INCLUDED	0.02	0.00096	39	4	7
4	14_105415346	AHNAK2	1.162	p.Thr2148Ala		AHLQGDLTLANKDLTAKDSRFKM	EXACT	PARTIAL	0.002	0.00914	31	3	2
5	1_17083776	MST1L	34.35	p.Arg674Leu		ARSRWPAVFTLVSVFVDWIHKVMRLG			0.0001	0.00578	54	3	6
6	6_168366581	MLLT4	11.744	p.Ser1708Ala	Driver	LPRDYEPPSPAPAPGAPPPPPQRNAS			0.0001	0.00054	80	3	3
7	8_52732961	PCMTD1	16.471	p.Pro342Thr		EPPQNLLREKIMKLTLPESLKAYLT	PARTIAL	PARTIAL	0.0008	0.00098	66	4	3
8	6_150001239	LATS1	3.182	p.Asp789Asn		KDNLYFVMDYIPGGNMMSLLIRMGIFPE	PARTIAL		0.0009	0.00126	58	3	7
9	3_123419461	MYLK	3.273	p.Asp952Asn	Passenger	RKVHSPQQVNFRSVLAKKGTSKT			0.001	0.01599	24	4	3
10	1_155697428	DAP3	12.728	p.Leu168Phe		IPDAHLWVKNCRDFLQSSYNKQRFD			0.002	0.00521	45	4	2
**154H, PDAC, 39 NON-SYNONYMOUS SOMATIC MUTATIONS**													
1	2_85576579	RETSAT	17.724	p.Arg309Trp		IAFHTIPVIQWAGGAVLTKATVQSVL	EXACT	EXACT	0.0004	0.00026	65	5	4
2	12_51453191	LETMD1	14.494	p.Asn367Asp		AELSLLLHNVVLLSTDYLGTRR	EXACT	EXACT	0.006	0.00298	50	4	4
3	20_34457413	PHF20	2.4	p.Arg288Gly		NSQTLQPITLELRRGKISKGCEVPL		EXACT	0.02	0.03246	19	4	2
4	3_57908703	SLMAP	3.553	p.Lys783Gln		KQSITDELQQCKNNLKLLREK			0.0007	0.00357	40	3	2
5	2_241700220	KIF1A	11.548	p.Ser769Phe		KKVQFQFVLLTDTLYFPLPPDLLPPEAA			0.0008	0.00201	72	5	5
6	2_238253286	COL6A3	16.075	p.Arg2459Trp		VAVVTYNNEVTTEIWFADSKRKSVLLDK			0.0009	0.00133	61	5	5
7	13_96592287	UGGT2	3.986	p.Val579Gly		KKDQNILTVDNVKSGLQNTF			0.002	0.01697	24	6	3
8	7_77256591	PTPN12	12.375	p.Gly532Glu	Driver	DRLPLDEKEHVTWSFHGPENAIPI	PARTIAL	PARTIAL	0.003	0.0391	12	6	1
9	18_55352319	ATP8B1	10.673	p.Asn486Lys		DHRDASQHKHNKIEQVDFSWNTYA			0.003	0.0339	14	5	2
10	8_9627645	TNKS	3.91	p.Gly1257Glu		HRQMLFCRVTLEKSFLQFSTMKMAHA	PARTIAL	PARTIAL	0.003	0.00039	39	6	5
**16AY, PDAC, 23 NON-SYNONYMOUS SOMATIC MUTATIONS**													
1	17_15134320	PMP22	11.978	p.Gly133Ser		HPEWHLNSDYSYSFAYILAWVAFPLALL	EXACT		0.0004	0.00018	86	4	8
2	X_54014354	PHF8	5.113	p.Ser621Tyr		LLMSNGSTKRVKSLYKSRRTKIAKKVDK			0.0001	0.0075	48	5	2
3	9_33794809	PRSS3	13983	p.Ser5Asn		MRETNVFTLKKGRSAPLVF			0.0004	0.00558	10	4	3
4	12_9085452	PHC1	9.639	p.Gln467Lys		TQQVPPSQSQQKAQTLVVQPMLQSSPL	PARTIAL		0.0004	0.0044	42	5	6
5	19_1037681	CNN2	20.083	p.Asp259Asn		APGTRRHIYDTKLGTNKCDNSSMSLQMG			0.0008	0.01696	27	5	5
6	20_50704942	ZFP64	1.539	p.Arg187Leu		YASRNSSQLTVHLLSHTGDTPFQ			0.002	0.00077	40	3	4
7	12_31254897	DDX11	5.459	p.Arg728His		LRQVHAHWEKGGLLGHLAARKKIFQE			0.003	0.00764	37	3	2
8	11_32954416	QSER1	3.78	p.Asn409Asp		SSNQQEVLSSVTNEDYPAQTRDLSSVSQ	PARTIAL		0.003	0.00478	39	3	4
9	1_27177681	ZDHHC18	4.114	p.His299Tyr		FFSIWSILGLSGFYTYLVASNLTTNEDI	PARTIAL		0.005	0.00012	106	4	8
10	20_60892518	LAMA5	23.906	p.Arg2465Gln		AKEELERLAASLDGAQTPLLQRMQT	PARTIAL	PARTIAL	0.008	0.00763	41	5	6

*The position of the mutation in the long peptide is indicated in red. ipMSDB HLA-I and ipMSDB HLA-II columns show the matching of the WT counterpart of the predicted neoantigen in the ipMSDB. Prediction of driver genes and mutation status annotations are derived from IntOGen database*.

### DC Vaccine Production

**Figure 4:**
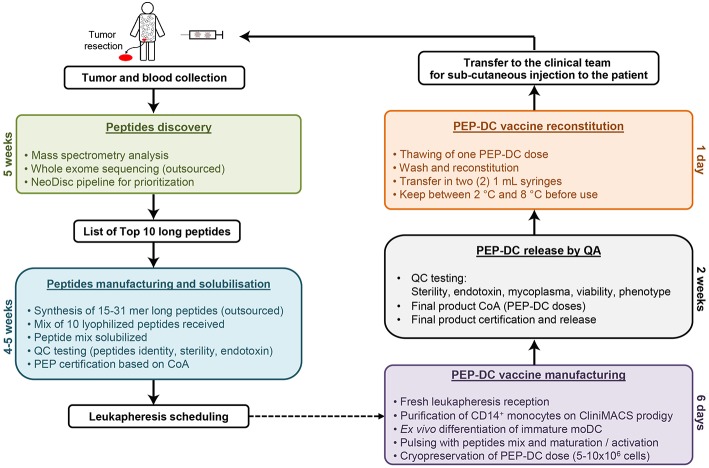
Schematic overview of PEP-DC manufacturing process and timelines.

The DC-vaccine used in this study is a frozen suspension of patient-specific, *ex vivo* cultured autologous monocyte derived DCs loaded with synthetic neoantigen (personalized) peptides. The proprietary name for the biological product comprising this substance is PEP-DC, which refers to Personalized Peptides loaded onto autologous DCs. Manufacturing of PEP-DC consists of five main steps described in Figure 4, starting with peptides identification and manufacturing. We have tested and validated this production process with healthy donor leukapheresis and peptides mixes of up to 9 peptides. Indeed, because of the peptide length, some synthesis failure should be expected even after sequence optimization and careful peptide selection. Based on three experimental batches, we can evaluate that PEP-DC process leads to the production of 2.8 ± 2.1 × 10^8^ PEP-DC cells, corresponding to 56 ± 41 PEP-DC cryopreserved vaccine doses (based on 5.0 × 10^6^ cells per dose) per manufacturing run. Specifications of the final product ensure safety (sterility, mycoplasma, endotoxin), viability (Trypan blue exclusion), identity (phenotype), and functionality (IL12p70 secretion upon maturation) of the PEP-DC vaccine (Table 2). For each vaccine injection, a PEP-DC dose is thawed, washed and reconstituted NaCl-Albumin before injection. Viability is checked on each reconstituted dose with a target of ≥60.0% viable cells. All PEP-DC batches prepared in this pilot study met specification for product release as described in Table 2. This confirms that our GMP-compliant manufacturing process is suitable for the production PEP-DC.

**Table 2:** Specification for release for the final product of PEP-DC doses.

	**Test**	**Analytical procedure**	**Specification**
PEP-DC at day 6 (final product)	Sterility	BacTEC (aerobic and anaerobic)	No growth
	Mycoplasma	MycoSeq	Negative
	Endotoxin	Endosafe	≤ 10.0 EU/mL
	Cell count	Manual cell count by Trypan blue exclusion	≥45.0 × 10^6^ viable cells
	Viability		≥60.0% viability
	Cell purity/identity	Flow cytometry	≥60.0% live HLA-DR^+^CD86^+^ cells ≤20.0% CD14^+^ cells
Culture supernatant at day 6 after maturation	Functionality IL-12p70	ELISA	≥50.0 pg/mL

### Pre-immunization Immunogenicity of PEP Candidates

**Figure 5:**
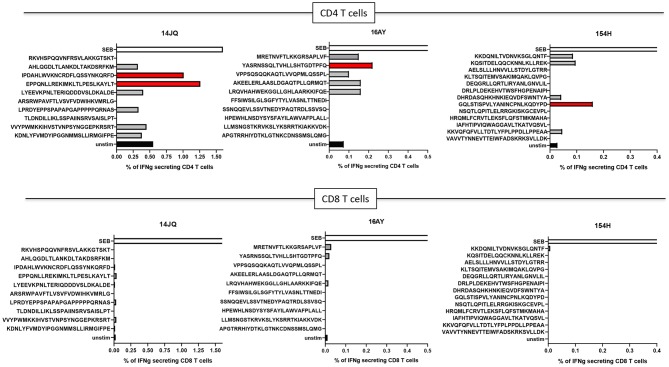
T-cell responses in donors 14JQ, 16AY, and 154H against long peptides. The percentage of IFN-γ-producing T cells are shown. Black (unstim) and white (SEB) bars represents negative and positive controls, respectively. Positive peptides are identified in red.

Even though immune responses against neoantigens prior to vaccination are typically rare, we decided to test if any of the 10 PEP long peptides may be recognized by autologous T cells from peripheral blood. Pre-immunization immunogenicity was tested for the three PDAC patients. Although some of the long peptides failed the synthesis quality control and could not be tested, CD4+ T-cell responses against PEP candidates were detected against at least one long peptide in all three donors, while no CD8+ T cell responses could be detected (Figure 5).

## Discussion and Innovation

Over the last 10 years immunotherapy has changed the treatment landscape of several tumor types in metastatic setting. Management of patients with non-metastatic cancer relies on multimodality treatment that includes surgical resection depending on tumor type and peri-operative chemotherapy. In PDAC, despite these aggressive measures, the high propensity of relapse has a detrimental effect on survival. The high metastatic potential is due to the presence of micrometastasis at systemic sites in patients with early-stage pancreatic cancer (83). In this proof of concept trial, our aim is to demonstrate that in such very aggressive diseases, there is a need to act without any delay, and early immunomodulation may be the key response.

Although pancreatic cancer patients present high frequencies of functional tumor-reactive T cells in the bone-marrow and blood (84), and show an average mutation burden similar to other solid tumors (85), parsing tumor immune microenvironment (TME) of pancreatic cancer seems to be a challenge. In PDAC, tumor-specific CTLs become “trapped” in the peritumoral tissue and in the tumor stroma, not reaching pancreatic tumor cells in sufficient amounts (86, 87). Additionally, exhaustion of effector CD8+ T-cells by the TME as well as hampered recruitment of cDC1s by downregulating CCL4 signaling upon constitutively active β-catenin signaling may explain the ineffective antitumoral response, which underscores the importance of endogenous DCs for initiating anti-tumor immunity.

### Rationale for Combination Immunotherapy in PDAC

Currently, clinical benefit using different agents in monotherapy is very limited in PDAC. Therefore, combination strategies are required, in order to obtain a synergistic effect on potential efficacy, yet keeping expected adverse events under manageable conditions. Therefore, it is important to establish both the scientific rationale of the proposed combination, as well as the best timing for introducing each component.

Treatment of metastatic cancer essentially relies on cytotoxic drugs that kill tumor cells or hinder their proliferation. Although a primary goal of anti-cancer chemotherapy is the tumor mass reduction, it is now clear that off-target effects, especially directed to the host immune system, may reduce the immunosuppressive activity of malignant cells and cooperate for successful tumor eradication (88). Gemcitabine (GEM) is a chemotherapeutic agent acting as a nucleoside analog that also targets ribonucleotide reductase by inactivating the enzyme irreversibly. It is used in various carcinomas such as non-small cell lung cancer, pancreatic cancer, bladder cancer, and breast cancer, and it represents the primary systemic agent for the treatment of pancreatic cancer. On standard dose schedules in patients with pancreatic cancer, the drug is associated with manageable toxicity, and its administration has led to a survival benefit both in the primary and adjuvant settings (89, 90). In advanced pancreatic cancer patients, GEM therapy may decrease memory T-cells, promote naive T-cell activation (91), and induce the proliferation of CD14^+^ monocytes and CD11c^+^ DC (92). GEM is also able to induce apoptotic destruction of tumor cells and potentially load the immune system with large amounts of tumor antigen, but this is not enough to initiate a protective antitumor response and adjuvant immunotherapy is required (93).

A peptide cocktail vaccine OCV-C01 containing epitope peptides [coding for vascular epithelial growth factor receptor (VEGFR1 ad VEGFR2)] was investigated in combination with GEM in the adjuvant treatment for resected pancreatic cancer patients (*n* = 30) in a single arm multicenter Phase II study. OCV-C01 combined with GEM was tolerable with a median DFS of 15.8 months (and a DFS rate at 18 months of 34.6%), which was favorable compared with previous data for resected pancreatic cancer (94). In another phase I pilot study, a Wilms tumor gene-1 peptide-pulsed DC vaccination was evaluated in combination with GEM as a first-line of treatment in 10 patients with advanced pancreatic cancer. WT1 peptide-pulsed DCGEM is feasible, well-tolerated, and effective for inducing anti-tumor T-cell responses (95). Kimura et al. evaluated a DC-based vaccine alone or in combination with lymphokine-activated killer (LAK) cells, along with gemcitabine and/or S-1 in 49 patients with inoperable pancreatic cancer (96). Of these patients, two manifested a complete remission, five a partial remission, and 10 had stable disease. The median survival of these individuals was 360 days, which appeared to be longer than what could be achieved with gemcitabine and/or S-1. Thus, the combination of DC-based immunotherapy and chemotherapy seems well-tolerated by advanced PDAC patients but warrants further investigation through combination with ICB or other immunotherapies. In our study, we build on the gemcitabine/capecitabine backbone for not fit pancreatic cancer population (ECOG PS 1 or 2) and explore the additive benefits of DC-vaccination from the 5th cycle of chemotherapy, followed by nivolumab treatment.

In pancreatic cancer, a possible explanation for the therapeutic failure of PD-1/PD-L1 blockade therapy is the lack of natural infiltration of effector immune cells in most cases (17, 18, 20). Vaccine-based immunotherapy is a potential strategy to activate effector T cell trafficking into the TME. Additionally, it has been shown that the repertoire of clonally expanded tumor antigen-reactive cells within TILs expresses PD-1 (97), either in spontaneous responses or vaccine-mediated. Furthermore, vaccination induces intratumoral PD-L1 expression (98), suggesting a role for PD-1 blockade in enhancing vaccine efficacy (98, 99). Consistently, in a preclinical model for pancreatic cancer, GVAX administration (a cancer vaccine composed of allogeneic pancreatic tumor cell line engineered to secrete GM-CSF) induced upregulation of PD-L1 expression when compared to untreated human and mouse pancreatic tumors. Combination therapy with GVAX and PD-1/PD-L1 blockade improved survival, and correlated with increased CD8+ T infiltration into pancreatic tumors (100).

Currently very few clinical trials combining cancer vaccines and PD-1/PD-L1 blockade have been reported in the setting of pancreatic cancer. Combination strategies using DC vaccines with ICB should generate an additive effect (98, 99, 101), with low additional toxicity due to DC vaccination (102, 103). Nesselhut et al. demonstrated that the efficacy of DC based therapy can be improved by blockade of PD-L1, enhancing the T-cell specific response (104). Dose and schedule for anti-PD-1 therapy and vaccines have been minimally studied; however, both PD-1 on activated T cells and PD-L1 on tumors appear rapidly following exposure to interferon (105), suggesting that early application of PD-1 blockade may be important. For this reason, we have decided to start nivolumab treatment 3 weeks after the end of SOC treatment, aiming also to avoid potential toxicities due to combined chemo-ICB.

Because Treg may persist despite checkpoint blockade, Treg depletion in conjunction with checkpoint blockade and vaccination may enhance clinical anti-tumor efficacy. Systematic reviews of the results of aspirin in cardiovascular studies have suggested that low-dose aspirin reduces overall cancer incidence and mortality including in pancreatic cancer (106, 107). In terms of its mechanism (108), it has been shown that non-steroidal anti-inflammatory drugs may limit carcinogenesis and enhance the immune response by (a) preventing prostaglandin E_2_ (PGE_2_)-mediated inhibition of DCs and reducing the transition of monocytes to immunosuppressive MDSCs (109); (b) reducing the inhibitory potential of Tregs induced by PGE2 (110); and (c) abrogating the PGE2 induced suppression of effector T-cell proliferation by regulatory T cells (111), therefore contributing to enhanced immune surveillance. Furthermore, PGE2 inhibitors like aspirin can counteract the FasL mediated elimination of activated lymphocytes by the tumor endothelial cells, as well as reduce the immunosuppressive conditions, thus enhancing the immune response against the tumor. We therefore consider that blockade of PGE2 in cancers using aspirin can reverse the endothelial barrier and synergize with vaccination allowing T cell infiltration. Consequently, we will use aspirin all along our study, which we expect to synergize with T cell activation by PD-1/PD-L1 blockade.

### Neoantigen Prediction and Selection for PEP-DC

Identification and selection of targets for neoantigen based vaccines is challenging. Mass spectrometry has been instrumental for the identification of cancer-associated antigens among the endogenously presented peptides. In recent years, dedicated computational pipelines for proteogenomic applications facilitated the direct identification of neoantigens by MS in murine and human cancer cell line models (49, 112–115), B cell lymphomas (116), and melanoma tissues (72) as well as other cryptic peptides resulting from unconventional coding sequences in the genome (117, 118). However, only a handful of neoantigens have been identified by MS in a given sample, and typically in high mutational load tumors such as melanoma (72). Indeed, we could not identify with discovery MS-based immunopeptidomics neoantigens in the three investigated PDAC samples. While several tumor-associated antigens were identified, after literature mining we concluded that these antigens might be poorly immunogenic, and in these three cases we decided to exclude non-mutated targets.

The prioritization and selection of neoantigens for personalized vaccines in low mutational load tumors like PDAC is largely performed with HLA ligand interaction prediction algorithms. The performance of such tools has improved significantly with the incorporation of MS HLA ligand elution data in the training of the algorithms, both for HLA-I (81, 119–121), and more recently for HLA-II (68). Furthermore, interrogation of properties of the thousands of different source-proteins has revealed biological determinants that correlate with presentation, such as level of translation and expression, turnover rate, proteasomal cleavage specificities, hotspots, and biological functions. Integrating such variables into a single predictor further improves prediction of neoantigens (70, 72, 120, 122). Because predictors of immunogenicity are still immature (123) false positives are inevitably included among the predicted neoantigens, which may eventually be included in a vaccine.

A main innovative aspect of our study is the identification of PDAC mutated neoantigens. We have designed NeoDisc, a novel proteogenomics antigen discovery pipeline for identification and selection of neoantigens, and we apply it for the first time in PDAC. NeoDisc integrates multiple state of the art prediction tools, large-scale ligandomic database, and a unique personalized and optimized design of long peptides that maximizes the likelihood that the selected mutations will eventually be presented by the HLA-I and HLA-II complexes on the loaded DCs. While in this PEP-DC study the existence of pre-existing immune responses against the long peptides is not a prerequisite for inclusion in the vaccine, such analysis is performed as part of a large translational program that aims to provide extensive immunogenicity training data that will allow future development to improve the performance of NeoDisc.

This proof of concept study aimed to assess specifically the feasibility of prioritizing immunogenic neoantigens with NeoDisc. Indeed, we were able to confirm for the three patients pre-existing immune responses against in total four long neoantigen peptides with autologous peripheral CD4+ T cells. No CD8+ T cell responses could be detected. This might be related to the low frequency of neoantigen specific CD8+ T cells. Alternative strategies could have been more sensitive to detect CD8+ T-cell responses, such as peptide-MHC multimers screening. However, unfortunately, there were no PBMC left to test this hypothesis. The clinical trial has not started yet, and therefore the investigation of immune responses post-vaccine could not be performed. This trial will give us the opportunity to (1) better understand PDAC TME since we will be able to evaluate the mutational rate in PDAC and predict the presentation of neoantigens; (2) assess the frequency of specific T cells to such mutant epitopes in PDAC patients, before and after treatment with ICB; (3) validate the immunogenicity of neoantigens and their therapeutic effect.

In conclusion, PDAC in early-stage remains a deadly disease with limited treatment options and the development of novel strategies tailored to individual patients is the key. Our approach is focused particularly on patients with a borderline performance status or a comorbidity profile that precludes multiagent adjuvant therapy (type folforinox). In this context, we give the opportunity even in patients with the worst prognosis to have access to innovative therapies.

## Data Availability

The raw data supporting the conclusions of this manuscript will be made available by the authors. To respect patient confidentiality, access to the data will be obtained by formal application to a Data Access Committee that requires researchers to sign a Data Access Agreement (DAA).

## Author Contributions

MB-S is responsible for the development of NeoDisc and for clinical antigen discovery, and wrote the manuscript. BS and FH developed NeoDisc. JM and HP performed Bristol-Myers Squibb (BMS) immunopeptidomics experiments. JR and DG developed MixMHC2perd. CS, DW, and ND provided the pancreatic cancer specimens and reviewed the manuscript. AD, KB, and SM contributed to the clinical trial design and manuscript writing. GC contributed to the clinical trial design. A-CT and AH developed the immunogenicity validation data. CB developed the vaccine clinical grade data. LK developed the clinical grade vaccine, conceived the clinical study, and wrote the manuscript.

### Conflict of Interest Statement

The authors declare that the research was conducted in the absence of any commercial or financial relationships that could be construed as a potential conflict of interest.

## Supplementary Material

Table S1 General clinical information and high resolution HLA typing.

Table S2 List of HLA-I and HLA-II peptides identified by MS-based immunopeptidomics in the three PDAC tumor samples, including MS intensity, peptide length and mass, identification score and posterior error probability (PEP), source proteins and gene names.
