# Supplementary material for: A Phase Ib Study of the Combination of Personalized Autologous Dendritic Cell Vaccine, Aspirin, and Standard of Care Adjuvant Chemotherapy Followed by Nivolumab for Resected Pancreatic Adenocarcinoma—A Proof of Antigen Discovery Feasibility in Three Patients
Source: Front Immunol. 2019 Aug 8;10:1832. doi: 10.3389/fimmu.2019.01832 (PMC6694698; doi:10.3389/fimmu.2019.01832)
Supplement: Table S1 — General clinical information and high resolution HLA typing. [file Data_Sheet_1.PDF]

Code	Diagnosis	Gender	Age	Stage	Resection R0/R1	Grade	HLA-I typing	HLA-I typing
14JQ	PDAC	M	46	pT2 pN1 (2/27) L1 V0 Pn1, stage IIB	R0	3	A:03:01<br>A:32:01<br>B:07:02<br>B:47:01<br>C:07:02<br>C:07:18	DPA1:01:03<br>X<br>DPB1:04:02<br>DPB1:23:01<br>DPB1:105:01<br>DPB1:138:01<br>DQA1:03:01<br>DQA1:05:05<br>DQB1:03:01<br>DQB1:03:02<br>DRB1:04:04<br>DRB1:11:01<br>DRB3:02:02<br>DRB4:01:03
154H	PDAC	M	48	pT3 pN1 (3/40) L1 V1 Pn1, stage IIB	R1	2	A:02:05<br>A:24:02<br>B:07:02<br>B:18:01<br>C:07:01<br>C:07:02	DPB1:04:01<br>X<br>DQA1:01:02<br>DQA1:05:05<br>DQB1:03:01<br>DQB1:06:02<br>DRB1:11:01<br>DRB1:15:01<br>DRB3:02:02<br>DRB5:01:01
16AY	PDAC	F	70	pT2 pN0 (0/28) L1 V0 Pn1, stage IB	R0	2	A:01:03<br>A:03:01<br>B:07:02<br>B:73:01<br>C:07:02<br>C:15:05	DPB1:04:01<br>DPB1:04:02<br>DPB1:126:01<br>DPB1:105:01<br>DQA1:01:02<br>DQA1:03:03<br>DQB1:02:02<br>DQB1:06:02<br>DRB1:04:05<br>DRB1:15:01<br>DRB4:01:03<br>DRB5:01:01

Supplementary Table 1. General clinical information and high resolution HLA typing.
